# N20-P25 Amplitude can Predict Awakening from Coma

**DOI:** 10.1007/s12028-025-02335-9

**Published:** 2025-08-11

**Authors:** Li Huang, Zhi-han Li, Mei-lin Ai, Qi Liu, Qian-yi Peng, Mi-lin Peng, Chun-guang Zhao, Li-na Zhang

**Affiliations:** 1 Department of Critical Care Medicine, Xiangya Hospital, Central South University, Changsha, Hunan Province China; 2 Hunan Provincial Clinical Research Center for Critical Care Medicine, Xiangya Hospital, Central South University, Changsha, Hunan Province China; 3 National Clinical Research Center for Geriatric Disorders, Xiangya Hospital, Central South University, Changsha, Hunan Province China

**Keywords:** Short-latency somatosensory evoked potential, Amplitude, Outcome

## Abstract

**Background:**

The objective of this study was to evaluate the relationship between the N20-P25 amplitude of short-latency somatosensory evoked potentials (SSEPs) and neurologic outcomes in patients in a coma state.

**Methods:**

We retrospectively enrolled neurocritical patients who were older than 18 years; were admitted to the Department of Critical Medicine, Xiangya Hospital, Central South University, from January 2017 to January 2021 for 1–3 days; had a Glasgow Coma Scale score ≤ 8; had a body temperature ≥ 35 °C; and had SSEP records. Good outcome was defined as Cerebral Performance Category scores 1–3 at 1 year. The specificity and sensitivity of different SSEP patterns and amplitudes were calculated.

**Results:**

A total of 457 patients were included in this study. The N20-P25 amplitude can be used for predicting awakening for traumatic brain injury (TBI) (area under the curve [AUC] 0.70, *p* = 0.0077), aneurysmal subarachnoid hemorrhage (SAH) (AUC 0.69, *p* = 0.005), intracerebral hemorrhage (ICH) (AUC 0.69, *p* = 0.005), and cardiac arrest (CA) (AUC 0.72, *p* = 0.0077). An N20-P25 amplitude > 1.6 μV predicted awakening in CA, with a sensitivity of 100% (95% confidence interval [CI] 81.6–100%) and specificity of 46.7% (95% CI 30.9–60.9%). In SAH, an N20-P25 amplitude > 0.74 μV predicted the sensitivity and specificity of awakening were 100% (95% CI 93.8–100%) and 16% (95% CI 8.3–28.5%), respectively. In TBI, an N20-P25 amplitude > 1.20 μV predicted awakening with a sensitivity of 100% (95% CI 86.2–100%) and a specificity of 34.2% (95% CI 21.2–50.1%). An N20-P25 amplitude > 0.65 μV predicted the sensitivity and specificity of awakening in ICH were 100% (95% CI 91.0–100%) and 14.3% (95% CI 5.7–31.5%), respectively.

**Conclusions:**

N20-P25 amplitude can predict awakening in patients in a coma state at 1 year. Different diseases have different cutoff values for predicting awakening.

**Supplementary Information:**

The online version contains supplementary material available at 10.1007/s12028-025-02335-9.

## Introduction

In the intensive care unit (ICU), many neurocritical patients are still in a coma after treatment for the primary injury. At present, multimodal methods, including clinical examination (Glasgow Come Scale Motor Response and brainstem reflex), Electroencephalogram (EEG), short-latency somatosensory evoked potential (SSEP), biological (neuron-specific enolase, S100 calcium binding protein *β*) and neuroradiological (computed tomography, magnetic resonance imaging) methods, are used to predict the neurologic outcome [1]. In developing countries, SSEPs can be used to evaluate the outcome of patients in a coma state because they can be monitored in the early stage, at the bedside, objectively, noninvasively, and efficiently [2]. In addition, SSEPs can be used to assess the prognosis of cardiac arrest (CA) under sedation and targeted temperature management and are less confounded by sedation and body temperature than EEG [3]. At present, SSEPs are widely monitored in domestic clinical practice, especially during surgery and in the ICU.

Previous studies have mostly dichotomized N20 presence and N20 absence to evaluate outcomes. However, the combination of absent and low-amplitude N20 appears to be highly predictive of a poor outcome, with higher sensitivity, but the cutoff values for N20 differ across studies [4–10]. However, research on the ability of SSEPs to predict good neurologic outcomes is in its infancy, and the conclusions are inconsistent. In neurocritical patients with conditions other than CA, such as traumatic brain injury (TBI) [11], aneurysmal subarachnoid hemorrhage (aSAH) [12], intracerebral hemorrhage (ICH) [13], and acute ischemic stroke (AIS) [14], the neurologic outcome is mostly predicted by a dichotomy (N20 present or absent, and the latency is normal or prolonged). However, studies on the prediction of neurologic outcomes by amplitude are lacking.

The aims of this study were to observe the predictive effect of the N20-P25 amplitude on the neurologic outcome in different neurocritical patients in a coma state, especially patients without CA, to observe the predictive effect of the N20-P25 amplitude on the outcome of different neurocritical patients at 1 year and calculate cutoff values for predicting different neurologic outcomes. This study is the first to use the N20-P25 amplitude to predict the neurologic outcome of neurocritical patients without CA. We assume that the SSEP amplitude is less than a value to predict a poor neurologic outcome and greater than a value to predict a good neurologic outcome.

## Methods

### Study Design, Patients, and Data Collection

This retrospective study included prospectively collected data from the Department of Critical Medicine, Xiangya Hospital, Central South University, from January 2017 to January 2021. We included patients who were older than 18 years, had a Glasgow Coma Scale (GCS) score ≤ 8, had a body temperature ≥ 35 °C, and had SSEP records (with or without sedation and analgesia), including CA, aSAH, ICH, AIS, and other disease (intracranial space-occupying surgery and sepsis-associated encephalopathy (SAE). Baseline data of patients, such as sex, age, length of ICU stay, GCS score, type of disease, type of sedatives and analgesics (including propofol, midazolam, sufentanil, and remifentanil) and dose, Acute Physiology and Chronic Health Evaluation II score, and Sequential Organ Failure Assessment score, were collected. The dose of sedatives and analgesics is the cumulative dose before the SSEPs are recorded. The research protocol was approved by the Ethics Committee of Xiangya Hospital of Central South University, and informed consent was waived because of its retrospective nature.

### SSEP Recording

SSEPs were monitored using Nicolet (EDX). The parameters were as follows: the recording electrode, reference electrode, and ground electrode, which were placed according to the international EEG 10–20 system. The median nerve of the wrist was stimulated, stimulation intensity was adjusted to produce visible thumb twitches, and the cathode was placed between the palmaris longus tendon and the flexor carpi radialis tendon, which are 2 cm in length at the proximal end of the wrist fold. The anode was placed 2–3 cm away from the cathode. The ground electrode was placed 5 cm above the stimulation point, and the reference electrode was placed at FPz at the midpoint of the frontal pole. The recording electrodes were placed at CP3, CP4, Fz, Cv7, and Erb’s point on both sides: CP3 and CP4, 2 cm behind C3 and C4; Fz, the midpoint of the forehead; Cv, located at the seventh cervical spine; and Erb’s point, 1 cm above the midpoint of the opposite clavicle. For AgCl electrode placement, alcohol and scrub cream were used to degrease and exfoliate the local skin and scalp, and then an appropriate amount of conductive paste was applied to minimize resistance. The impedance of all electrodes was ≤ 5 kΩ. The bandpass was set as a high-pass filter below 3 Hz and a low-pass filter above 2,000 Hz. The analysis time was 50 ms. The duration of the stimulation square wave was 0.1–0.2 ms and could reach 0.5 ms if necessary. The stimulation frequency was 1–5 Hz. The amplifier sensitivity was 100 μV, with a display of 1 μV. At least two groups of 500 tests were performed on average.

### SSEP Definition

**Fig. 1:**
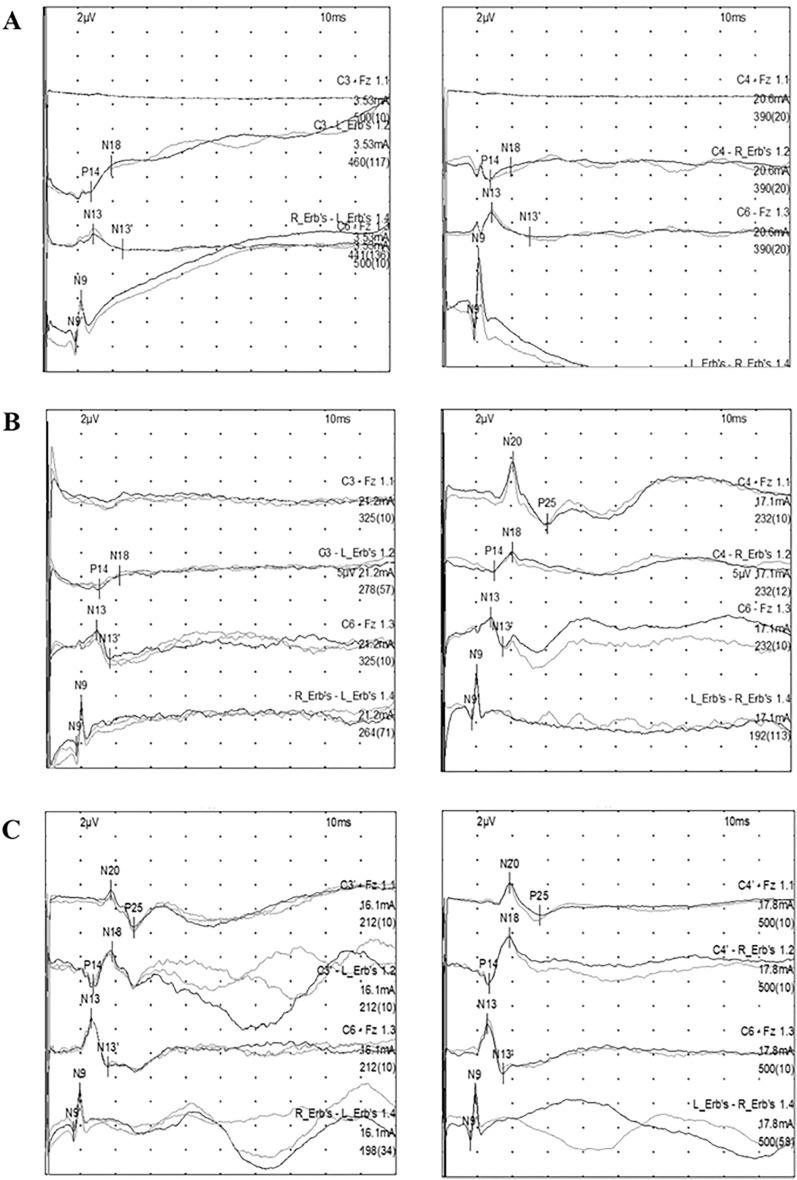
Pattern categories according to the presence of N20 on cortical somatosensory evoked potential recordings. **a** Bilaterally absent (*n* = 37). **b** Unilaterally present (*n* = 85). **c** Bilaterally present (*n* = 335). Erb’s, Erb’s point; Fz, frontal pole electrode; C3, C3 point; C6, C6 spinous process; C3' and C4', C3' and C4' point

All the SSEP recordings were reinterpreted by the authors (Li Huang and Mei-lin Ai; when they are inconsistent, they will be discussed with an experienced clinical neurophysiologist), who were blinded to patient outcomes. When normal potentials over Erb’s point (N9) and the cervical spinal cord (N13) were present, the SSEPs were analyzed. N20 is dichotomized into N20 present and absent according to the N20-P25 amplitude. “Bilaterally absent” is recorded as A/A, “unilaterally present” is recorded as A/P, “bilaterally present” is recorded as A/A, and A/P and P/P are defined as N20 present (Fig. 1). The N20-P25 amplitude was defined as the difference between the upward negative wave peak and the subsequent downward negative wave peak (N20-P25 peak-peak) approximately 20 ms after stimulation. The receiver operating characteristic (ROC) curves were plotted using the highest value and the average value of the two sides for patients with SSEPs, and the final amplitude was taken, which had the larger area under the curve (AUC). If the patient had multiple data points within 1–3 days, the highest record of using the patient under normal temperature (with or without sedatives and analgesia) was used. We excluded those with a peak-to-peak noise amplitude > 0.25 μV after averaging.

### Outcome Assessment

The neurologic outcomes of patients at 1 year after hospital discharge, as assessed by structured telephone interviews, were defined by the Cerebral Performance Category (CPC) scores. A good neurologic outcome was defined as CPC scores 1–3 (patients awakening), and a poor neurologic outcome was defined as CPC scores 4–5 (vegetative state or [brain] death). Investigators conducting neurologic outcome assessments were blinded to the SSEP records.

### Statistical Analysis

To describe the baseline data, SSEP data and the 1-year neurologic outcomes of the patients were used. The categorical variables are expressed as the number and percentage, and the continuous variables are expressed as the mean and standard deviation or the median and interquartile range (whether it conforms to a normal distribution). A scatter plot was constructed to show the N20-P25 amplitudes of different neurologic outcomes. For the two groups with different outcomes, differences were tested using a χ^2^ test for categorical variables and Student’s *t-*test for continuous variables, given a normal distribution, or the Mann‒Whitney *U-*test. ROC curves were used to evaluate the predictive effect of the N20-P25 amplitude on the neurologic outcome. The AUC was calculated, and the sensitivity and specificity of different N20-P25 amplitudes in predicting the neurologic outcome, including the 95% confidence interval (CI), were calculated. A logistic regression model was used to analyze variables related to outcomes and calculate the effect of the N20-P25 amplitude on outcomes after adjusting for other variables (age, length of ICU stay, type of disease, GCS score, the cumulative dose of sedatives administered before SSEPs). All the statistical analyses were performed using IBM SPSS version 26.0 (IBM Corporation, Armonk, NY). Figures were drawn using Prism 8.0.1 (GraphPad Software). All values were two-tailed, and a *p* value < 0.05 was considered significant.

## Results

### Baseline Characteristics of the Participants

**Fig. 2:**
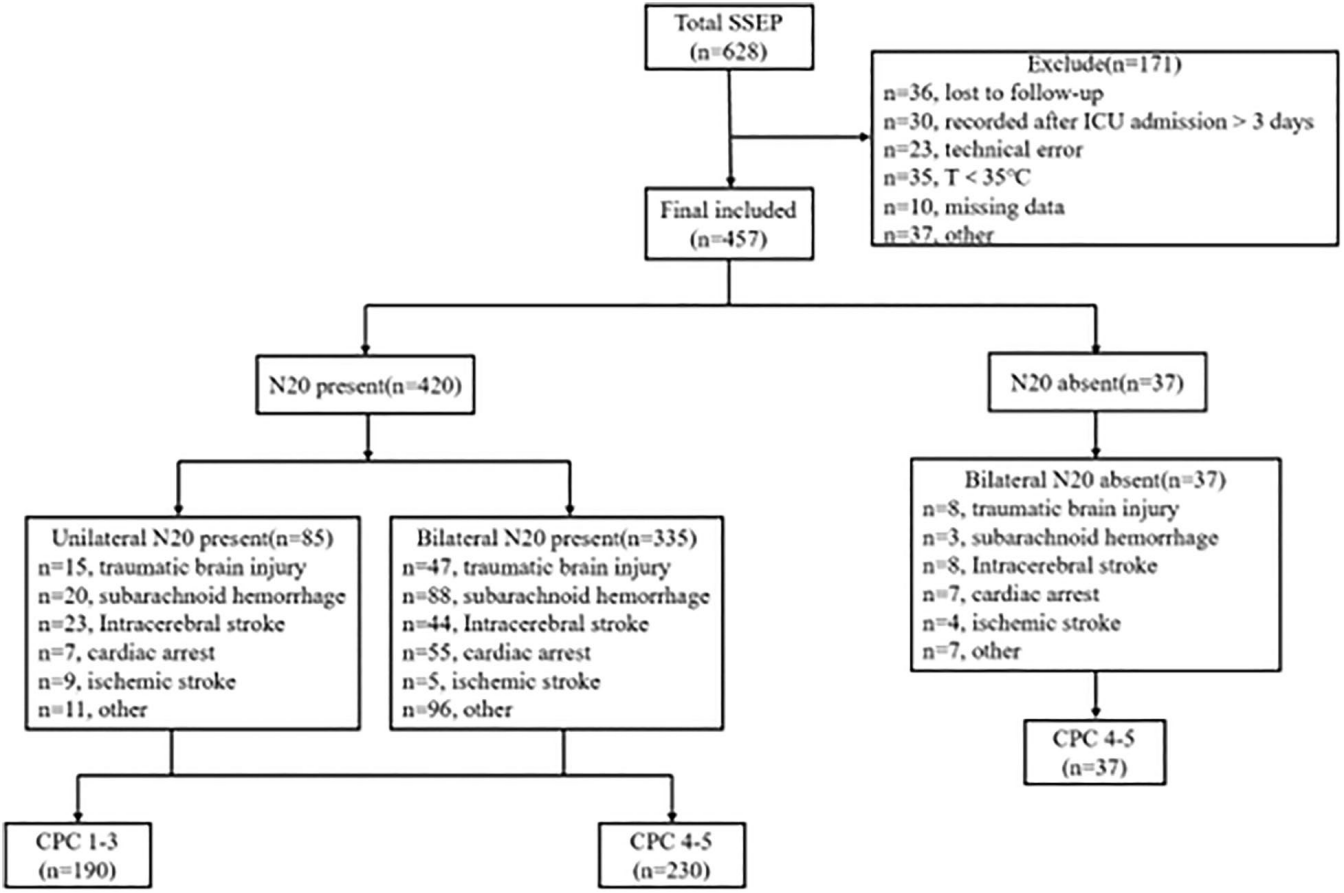
Flowchart. CPC Cerebral Performance Category, ICU intensive care unit, SSEP short-latency somatosensory evoked potential

A total of 628 adult neurocritical patients who had SSEP records and a GCS score ≤ 8 were admitted to the ICU of Xiangya Hospital of Central South University from January 2017 to January 2021. Among them, 36 patients were missed, 30 patients were recorded after ICU admission 3 days, 23 patients had technical errors, 35 patients had a target temperature 35 ℃, 10 patients had missing data, 37 patients had other reasons (e.g., did not have classical SSEP), and, ultimately, 457 patients were included in the analysis (Fig. 2). Age, length of ICU stay, GCS score, type of disease, and the cumulative dose of propofol administered before SSEPs were monitored were related to the neurologic outcome at 1 year (Table 1).

**Table 1:** Baseline characteristics of participants

	CPC 1–3 (*n* = 190)	CPC 4–5 (*n* = 230)	*p* value
Age, years, median (IQR)	54 (45–63)	60 (49–68)	< 0.001*
Male, n (%)	123 (64.7)	155 (67.4)	0.57
Length of ICU stays, days, median (IQR)	10 (5–18)	13 (7–21)	0.003*
GCS, median (IQR)	8 (4–8)	3 (3–6)	< 0.001*
Disease, n (%)			0.002*
TBI	24 (5.25)	38 (16.5)	
aSAH	58 (12.69)	50 (21.7)	
ICH	39 (8.53)	28 (12.2)	
CA	17 (3.72)	45 (19.6)	
Ischemic stroke	6 (1.31)	8 (3.48)	
Others^a^	46 (10.07)	61 (26.5)	
Medicine, median (IQR)			
Propofol, g	0.2 (0–1)	0 (0–0.5)	< 0.001*
Midazolam, mg	0 (0–108)	25 (0–150)	0.113
Sufentanil, μg	0 (0–0)	0 (0–0)	0.673
Remifentanil, mg	0 (0–3)	0 (0–4)	0.459

*aSAH* aneurysmal subarachnoid haemorrhage, *CA* cardiac arrest, *CPC* Cerebral Performance Category, *GCS* Glasgow Coma Scale, *ICH* intracerebral haemorrhage, *ICU* intensive care unit, *IQR* interquartile range, *TBI* traumatic brain injury

^*^p < 0.05

^a^Others includes intracranial space-occupying surgery and sepsis associated encephalopathy

## SSEP Findings

**Fig. 3:**
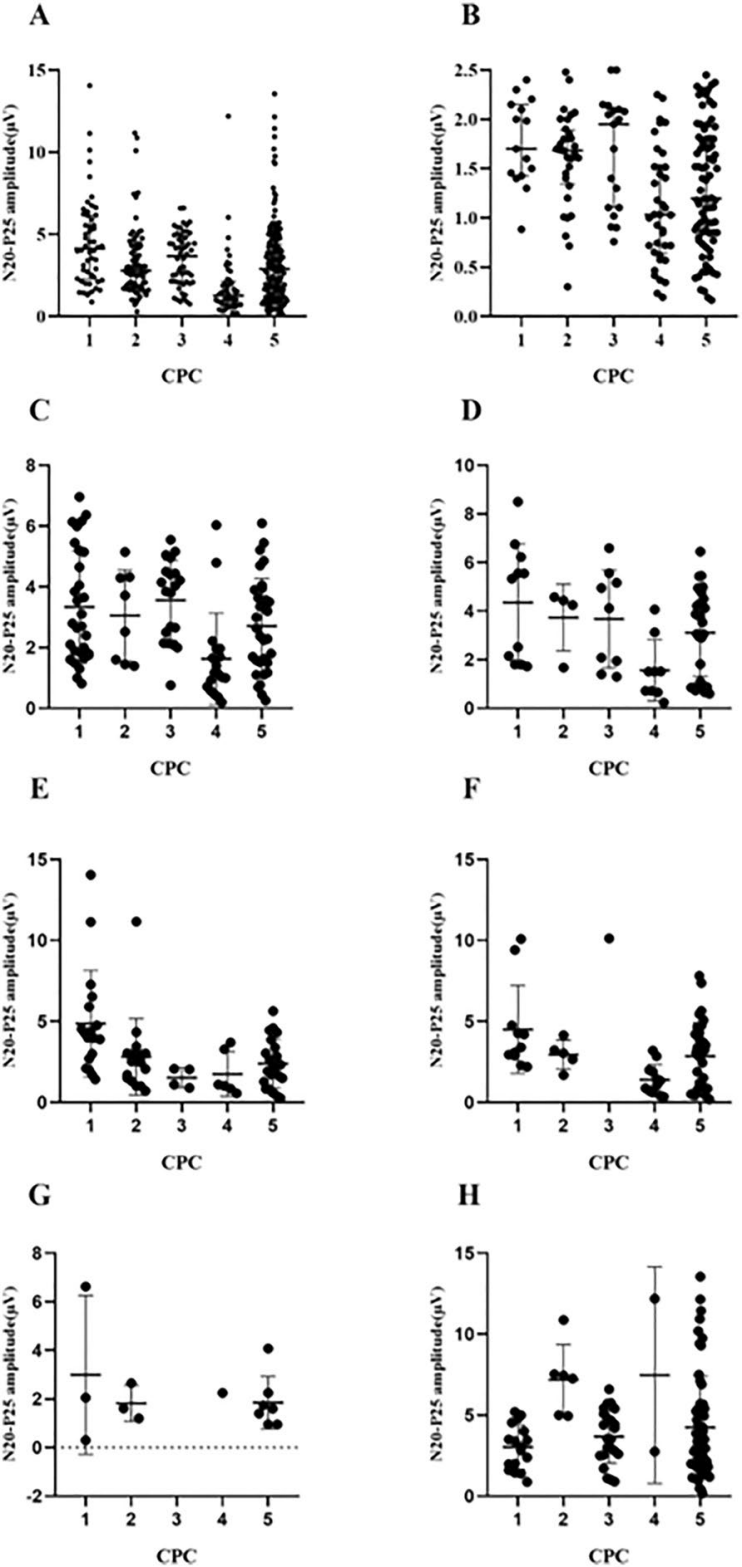
**a** Scatterplots for the N20-P25 amplitudes of the 420 patients with SSEPs present according to CPC score: CPC 1 (*n* = 55), CPC 2 (*n* = 79), CPC 3 (*n* = 56), CPC 4 (*n* = 48), CPC 5 (*n* = 182). **b** Y-axis restricted to low amplitudes (2.5 μV). **c** Scatterplots for the N20-P25 amplitudes of the patients with aSAH with SSEPs present according to CPC score: CPC 1 (*n* = 30), CPC 2 (*n* = 8), CPC 3 (*n* = 20), CPC 4 (*n* = 18), CPC 5 (*n* = 32). **d** Scatterplots for the N20-P25 amplitudes of the patients with TBI with SSEPs present according to CPC score: CPC 1 (*n* = 11), CPC 2 (*n* = 4), CPC 3 (*n* = 9), CPC 4 (*n* = 9), CPC 5 (*n* = 29). **e** Scatterplots for the N20-P25 amplitudes of the patients with ICH with SSEPs present according to CPC score: CPC 1 (*n* = 18), CPC 2 (*n* = 17), CPC 3 (*n* = 4), CPC 4 (*n* = 6), CPC 5 (*n* = 22). **f** Scatterplots for the N20-P25 amplitudes of the patients with CA with SSEPs present according to CPC score: CPC 1 (*n* = 11), CPC 2 (*n* = 5), CPC 3 (*n* = 1), CPC 4 (*n* = 12), CPC 5 (*n* = 33). **g** Scatterplots for the N20-P25 amplitudes of the patients with AIS with SSEPs present according to CPC score: CPC 1 (*n* = 3), CPC 2 (*n* = 3), CPC 3 (*n* = 0), CPC 4 (*n* = 1), CPC 5 (*n* = 7). **h** Scatterplots for the N20-P25 amplitudes of the other patients with SSEPs present according to CPC score. CPC 1 (*n* = 17), CPC 2 (*n* = 6), CPC 3 (*n* = 23), CPC 4 (*n* = 2), CPC 5 (*n* = 59). *AIS* acute ischemic stroke, *aSAH* aneurysmal subarachnoid hemorrhage, *CA* cardiac arrest, *CPC* Cerebral Performance Category, *ICH* intracerebral hemorrhage, *SSEP* short-latency somatosensory evoked potential, *TBI* traumatic brain injury

Among the 457 patients, 37 patients were considered A/A, 85 patients were considered A/P, and 335 patients were considered P/P. The baseline characteristics of the patients with N20 present (P/P, A/P) are shown in Table 1. A total of 190 patients had CPC scores 1–3 at 1 year, and 230 patients had CPC scores 4–5 at 1 year. The N20-P25 amplitude and CPC score scatter plots are shown in Fig. 3.

Among the 420 patients with SSEPs present, the ROC curves were made for CPC scores at 1 year and the average value or the highest value of N20-P25 amplitude. The AUC of “the average value of N20-P25 amplitude group” is 0.58 (95% CI 0.52–0.64, *p* = 0.0108). The AUC of “the highest value of N20-P25 amplitude group” is 0.54 (95% CI 0.48–0.60, *p* = 0.1808) (Fig. S1).

Among patients with SSEP present, 20 patients had SSEP on the first and third days of ICU admission, 13 patients had CPC scores 1–3, 7 patients had CPC scores 4–5 (Fig. S2), 42 patients had SSEP on the third day of ICU admission, and 358 patients had SSEP on the first day of ICU admission.

## SSEP Amplitudes and Outcomes

After adjusting for age, GCS score, length of ICU stay, and type of disease, each 1-μV increase in N20-P25 amplitude was associated with a decreased risk of poor neurologic outcome (odds ratio 0.74, 95% CI 0.66–0.82) (see Table 2).

**Table 2:** Logistic regression analysis

	*p* value	OR	95% CI
N20-P25 amplitude	< 0.0001	0.74	0.66–0.82
Age	< 0.0001	1.03	1.01–1.05
Length of ICU stay	0.001	1.05	1.02–1.09
GCS	< 0.0001	0.71	0.75–0.78
Disease	0.04	–	–
Propofol	0.02	0.77	0.61–0.970
Midazolam	0.16	–	–

*CI* confidence interval, *GCS* Glasgow Coma Scale, *ICU* intensive care unit, *OR* odds ratio

Among the 85 patients considered A/P, 25 patients had CPC scores 1–3, and 60 patients had CPC scores 4–5. The median N20-P25 amplitude of the group with CPC scores 1–3 was 2.05 μV, and the median N20-P25 amplitude of the group with CPC scores 4–5 was 0.85 μV; there was a statistically significant difference in N20-P25 amplitude between the two groups (*p* < 0.001) (Fig. S3).

**Fig. 4:**
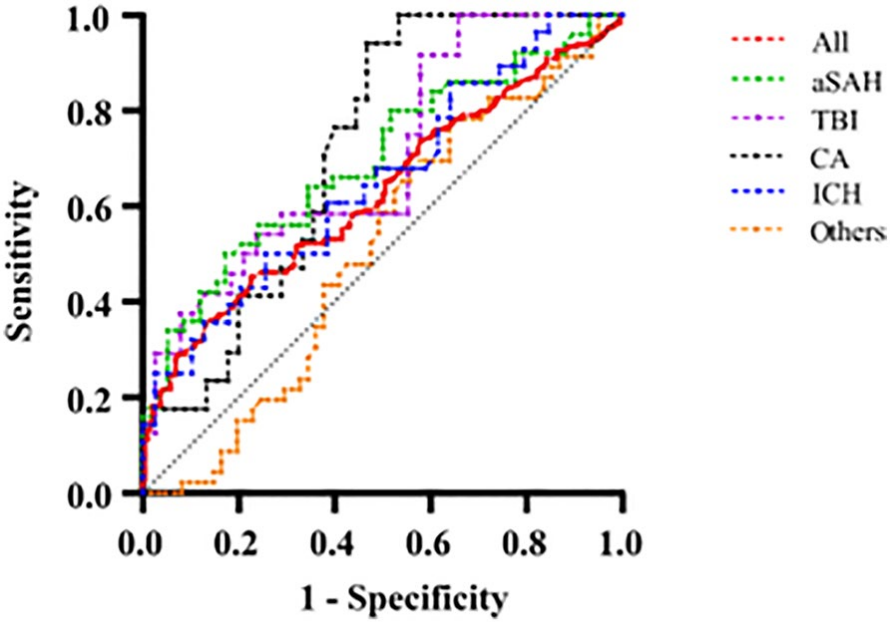
The receiver operating characteristic curve for Cerebral Performance Category at 1 year showing the predictive powers of SSEP amplitudes. Areas under the curve are as follows: all patients, 0.63 (95% CI 0.57–0.68, *p* < 0.0001); aSAH, 0.69 (95% CI 0.59–0.79, *p* = 0.005); TBI, 0.70 (95% CI 0.60–0.84, *p* = 0.0077); CA, 0.72 (95% CI 0.60–0.85, *p* = 0.0077); ICH, 0.65 (95% CI 0.52–0.79, *p* = 0.0343); others (intracranial space-occupying surgery and sepsis associated encephalopathy), 0.51 (95% CI 0.40–0.62, *p* = 0.8775). *aSAH* aneurysmal subarachnoid hemorrhage, *CA* cardiac arrest, *ICH* intracerebral hemorrhage, *SSEP* somatosensory evoked potential, *TBI* traumatic brain injury

Among the 420 patients with SSEPs present, the sensitivity of an N20-P25 amplitude < 0.88 μV for predicting a vegetative state or (brain) death was 18.3% (95% CI 13.8–23.8%), and the specificity was 97.8% (95% CI 94.5–99.2%). The sensitivity of an N20-P25 amplitude > 4.53 μV to predict awakening was 29.5% (95% CI 23.5–36.3%), and the specificity was 80% (95% CI 74.4–84.7%). ROC analysis revealed that CA, TBI, aSAH, and ICH had AUCs of 0.72 (95% CI 0.60–0.85, *p* = 0.0077), 0.70 (95% CI 0.60–0.84, *p* = 0.0077), 0.69 (95% CI 0.60–0.80, *p* = 0.005), and 0.65 (95% CI 0.52–0.79, *p* = 0.0343), respectively (see Fig. 4). In the AIS group (AUC 0.5417, *p* = 0.7963) and other disease group (AUC 0.5087, *p* = 0.877), the neurologic outcome could not be predicted using the N20-P25 amplitude.

### CA

For CA, 7 patients were considered A/A, 7 patients were considered A/P, and 55 patients were considered P/P. In 62 patients with SSEPs present, an N20-P25 amplitude < 1.12 μV predicted a vegetative state or (brain) death with a sensitivity of 35.6% (95% CI 23.3–50.2%) and a specificity of 100% (95% CI 81.6–100%). The sensitivity of an N20-P25 amplitude > 4.7 μV in predicting awakening was 23.5% (95% CI 10.0–47.3%), and the specificity was 86.7% (95% CI 73.8–93.7%). An N20-P25 amplitude > 1.6 μV predicted awakening with a sensitivity of 100% (95% CI 81.6–100%) and a specificity of 46.7% (95% CI 30.9–60.9%) (see Table S1).

## aSAH

For aSAH, 3 patients were considered A/A, 20 patients were considered A/P, and 88 patients were considered P/P. Among the 108 patients with SSEPs present, 58 patients had CPC scores 1–3, and 50 patients had CPC scores 4–5. An N20-P25 amplitude < 0.74 μV predicted a vegetative state or (brain) death with a sensitivity of 16% (95% CI 8.3–28.5%) and a specificity of 100% (95% CI 93.8–100%). In 28 patients with CPC scores 1–3 who had N20-P25 amplitudes > 3.64 μV, the sensitivity of N20-P25 amplitudes > 3.64 μV in predicting awakening was 48.3% (95% CI 35.9–60.8%), and the specificity was 80% (95% CI 67–88.9%). An N20-P25 amplitude > 0.74 μV predicted awakening with a sensitivity of 100% (95% CI 93.8–100%) and a specificity of 16% (95% CI 8.3–28.5%) (see Table S1).

### TBI

For TBI, 8 patients were considered A/A, 15 patients were considered A/P, and 47 patients were considered P/P. In 62 patients with SSEPs present, 24 patients had CPC scores 1–3 and 38 patients had CPC scores 4–5. An N20-P25 amplitude < 1.2 μV predicted a vegetative state or (brain) death with a sensitivity of 34.2% (95% CI 21.2–50.1%) and a specificity of 100% (95% CI 86.2–100%). Eleven patients with CPC scores 1–3 had N20-P25 amplitudes > 4.54 μV, and a N20-P25 amplitude > 4.54 μV predicted awakening with a sensitivity of 45.8% (95% CI 27.9–64.9%) and a specificity of 81.6% (95% CI 66.6–90.8%). Similarly, an N20-P25 amplitude > 1.20 μV predicted awakening with a sensitivity of 100% (95% CI 86.2–100%) and a specificity of 34.2% (95% CI 21.2–50.1%) (see Table S1).

### ICH

For ICH, 8 patients were considered A/A, 23 patients were considered A/P, and 44 patients were considered P/P. In 67 patients with SSEPs present, 14 patients had CPC scores 1–3 and N20-P25 amplitudes > 3.89 μV. The sensitivity of N20-P25 amplitudes > 3.89 μV in predicting awakening was 35.9% (95% CI 22.7–51.6%), and the specificity was 85.7% (95% CI 68.5–94.3%). An N20-P25 amplitude > 0.65 μV predicted awakening with a sensitivity of 100% (95% CI 91.0–100%) and a specificity of 14.3% (95% CI 5.7–31.5%). An N20-P25 amplitude < 0.66 μV predicted a vegetative state or (brain) death with a sensitivity of 14.3% (95% CI 5.7–31.5%) and a specificity of 100% (95% CI 91.0–100%) (see Table S1).

## Discussion

This retrospective study was conducted at Xiangya Hospital of Central South University to observe the relationship between SSEPs and neurologic outcomes. We found that after adjusting for sedatives (propofol, midazolam), length of ICU stay, age, GCS score, and disease type, the risk of poor neurological outcome decreased with increasing N20-P25 amplitude.

This study is the first to include patients with “unilateral presence” in the outcome analysis. In our clinical practice, we found that patients with “unilateral presence” had variable neurologic outcomes, so we speculate that there is a “dose-dependent” effect of unilateral N20-P25 amplitude on patient outcome (Fig S3). In this study, the ROC curves were plotted using the highest value and the average value of the two sides for patients with SSEPs, and the AUC of the average value was found to be larger than the AUC of the highest value (Fig S1). Withdrawal of life-sustaining treatment is not legal in our country, and for A/P, the average of value has a high sensitivity for predicting a good outcome and a high specificity for a poor outcome. Therefore, the average value of both sides was taken for the analysis of patients with SSEPs. Ultimately, we found that the N20-P25 amplitude can predict outcomes in all groups except for the AIS and other disease groups. Furthermore, the results of the AIS group may be highly biased due to the limited number of patients (*n* = 14). The other disease group had patients with postoperative intracranial occupancy and SAE, and there may be other factors affecting the neurologic outcome at 1 year. In addition, differences were found in N20 latencies between the two groups with different neurologic outcomes.

The specificity of previous studies for predicting poor outcomes in patients with CA was 100%, and the sensitivities of N20-P25 amplitudes below 0.64 μV [7], 0.4 μV [4], and 0.99 μV [9] were 74.5%, 11%, and 44%, respectively. N20 was less predictive of good outcomes than poor outcomes (low specificity and sensitivity) [4, 7, 8, 10, 15]. In this study, the cutoff values for predicting outcomes were greater than those in other studies, and further analysis revealed that the cumulative doses of sedatives used by patients were low (CPC scores 1–3: median propofol dose of 0.2 g and median midazolam dose of 0 mg; CPC scores 4–5: median propofol dose of 0 g and median midazolam dose of 20 mg), whereas all patients’ SSEP recordings were measured at normal temperature. Therefore, the related sedative and analgesic doses, SSEP stimulation parameters, and patient temperatures were considered. In addition, the inclusion of patients with unilateral N20 presence in the amplitude analysis of this study may also be one of the reasons for the different cutoff values.

In patients with aSAH, the outcome depends not only on the degree of primary injury but also on the degree of secondary injury caused by vasospasm, the inflammatory response, spreading depolarization, etc. Fewer studies have used SSEPs to predict long-term neurological outcomes [12, 16]. Because there is a strong correlation between electrophysiological changes and regional cerebral blood flow in the brain and when cerebral blood flow decreases to 14–16 mL/100 g/min, the amplitude of SSEPs can be reduced to 50% [17]. Therefore, the amplitude of SSEPs in patients with aSAH is mostly used for timely detection of intraoperative ischemia and postoperative secondary brain injury [18–21]. In this study, the sensitivity of an N20-P25 amplitude < 0.74 μV for predicting a poor outcome was 16%, and the specificity was 100%. The sensitivity of an N20-P25 amplitude > 3.64 μV for predicting a good outcome increased to 48.3%, and the specificity was 80%. An N20-P25 amplitude > 0.74 μV predicted awakening with a sensitivity of 100% and a specificity of 16%. Therefore, in the early stage of SAH, the neurological outcome can be preliminarily evaluated by the amplitude of SSEPs.

In patients with TBI, previous studies have noted that the absence of bilateral N20 may not provide the same strength of evidence of irreversible cortical injury as in patients with CA. Therefore, the prognostic impact of the absence of bilateral N20 on poor outcomes in patients with TBI is uncertain [13, 22, 23]. In our study, eight patients with bilateral N20 absence in the first 3 days were found to have poor neurologic outcomes, with a specificity of 100%. In addition, the amplitude was used for the first time to predict the outcome of patients with TBI, and the specificity of an N20-P25 amplitude < 1.2 μV to predict a poor neurologic outcome was 100%. An N20-P25 amplitude > 1.20 μV predicted awakening with a sensitivity of 100% and a specificity of 34.2%. The sensitivity increased to 45.8%, and the specificity decreased to 81.6% for N20-P25 amplitudes > 4.54 μV.

In patients with stroke, SSEPs are relatively poor at assessing outcomes. In addition, the relationship between SSEPs and outcomes is conflicting because of the different types and sites of stroke. Unlike patients with AIS and ICH, who present with diffuse brain damage early in CA, patients tend to have brain damage on the focal side first and contralateral involvement later. SSEPs have an asymmetrical bilateral presentation, with bilateral symmetrical abnormalities appearing only when the condition is further aggravated by whole-brain functional damage. Previous studies have used N20 absence to predict poor outcomes [24]. In this study, the sensitivity of predicting poor outcomes in patients with ICH with N20-P25 amplitudes < 0.66 μV was 14.3%, and the specificity was 100%. The sensitivity of an N20-P25 amplitude > 3.89 μV for predicting a good outcome was 35.9%, and the specificity was 85.7%. The sensitivity of an N20-P25 amplitude > 0.65 μV for predicting a good outcome was 100%, and the specificity was 14.3%. Statistical analysis of patients with AIS was not performed because of the small number of patients included.

The advantages of this study include the following: all patients underwent SSEPs at a normal temperature, and the effect of body temperature on SSEPs did not affect the results. This is the first quantitative analysis of patients with unilateral N20-P25 amplitudes, suggesting a new idea for future SSEP wave amplitude studies. The analysis of sedatives and analgesics in this study included the cumulative dose of drugs in the logistic regression, and the study did not dichotomize this variable on the basis of presence or absence. This study excluded the effects of sedative and analgesic doses on patients’ neurologic outcomes, observed the change in amplitude weight in the regression equation before and after the inclusion of sedative and analgesic doses in the regression analysis, and revealed that there was no change in N20-P25 amplitude weight before and after inclusion. Therefore, the effects of sedatives and analgesics on amplitude were also excluded. This study was the first to quantify the N20-P25 amplitude in patients with aSAH, ICH, and TBI to assess neurologic outcomes and revealed that the N20-P25 amplitude could also be used to predict neurologic outcomes in this group of patients with different cutoff values.

The limitations of this study include the following: this is a single-center retrospective study, and the generalizability of the results is limited. The sample size of each disease was small, and bias existed. The A/P model was used for the first time for amplitude analysis, and many clinical studies are needed to validate it. The clinicians were not blinded to the SSEP results, which may have interfered with treatment bias; the results of this study are inconsistent with those of previous studies and need to be interpreted with caution.

## Conclusions

N20-P25 amplitude can be used to predict awakening from coma within one year, especially in patients with aSAH, TBI, and ICH. It can be widely used in the early stage, at the bedside, objectively, noninvasively, and efficiently. Different diseases have different cutoff values for predicting the outcomes of different neurological functions.

## Supplementary Information

Below is the link to the electronic supplementary material.

Supplementary file1 (DOCX 172 KB)

Supplementary file2 (DOCX 20 KB)

## Data Availability

All data generated or analyzed during this study are included in this published article.
