# Supplementary material for: N20-P25 Amplitude can Predict Awakening from Coma
Source: Neurocrit Care. 2025 Aug 11;43(3):902–10. doi: 10.1007/s12028-025-02335-9 (PMC12647323; doi:10.1007/s12028-025-02335-9)
Supplement: Supplementary file 1 — Supplementary file1 (DOCX 172 KB) [file 12028_2025_2335_MOESM1_ESM.docx]

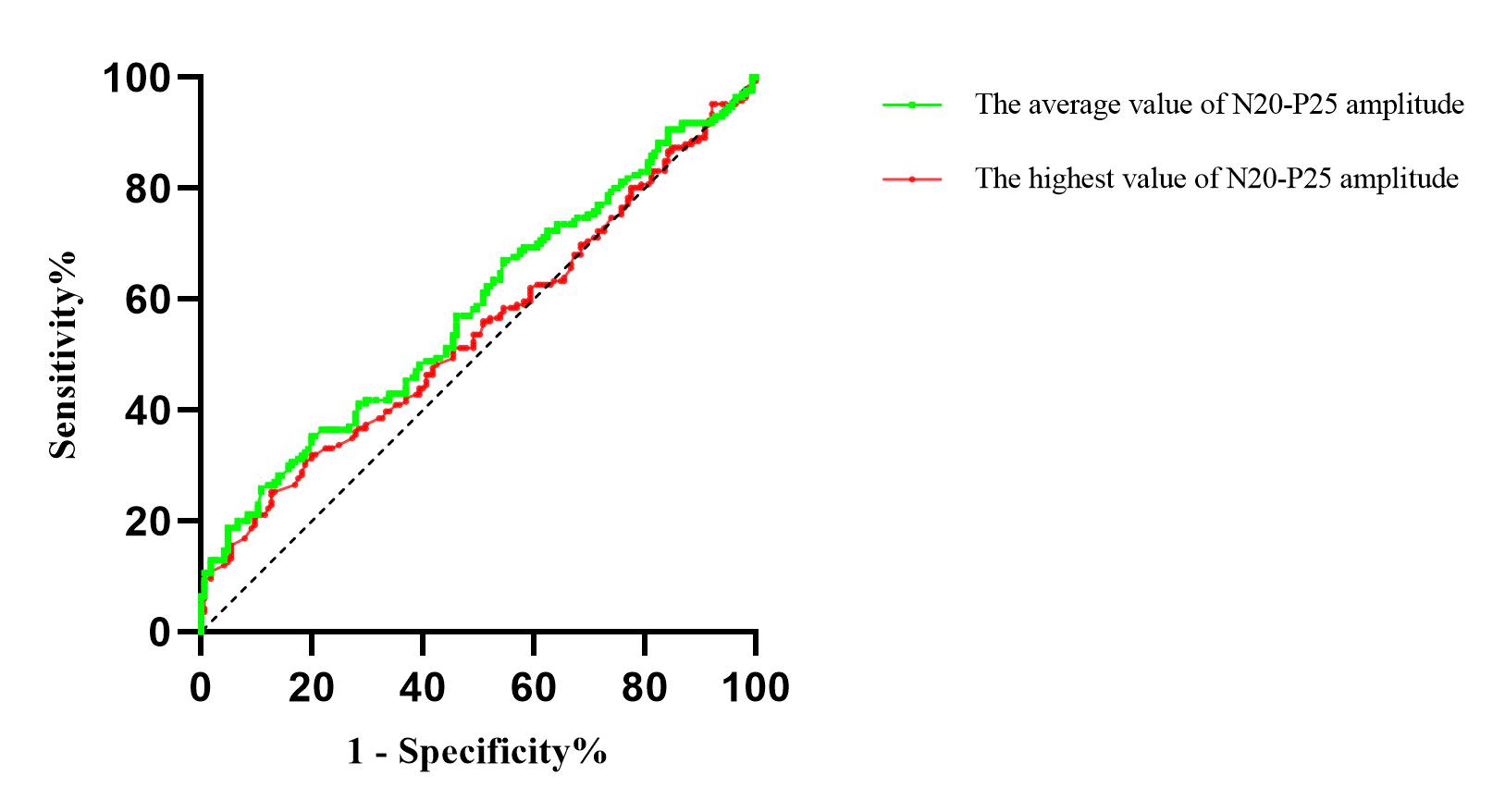


**Fig. S1.** The receiver operating characteristic (ROC) curves for Cerebral Performance Category scores at 1 years and the average value or the highest value of N20-P25 amplitude. The AUC of “The average value of N20-P25 amplitude group” is 0.58(95% 0.52-0.64, *p*=0.0108). The AUC of “The highest value of N20-P25 amplitude group” is 0.54(0.48-0.60, *p*=0.1808).


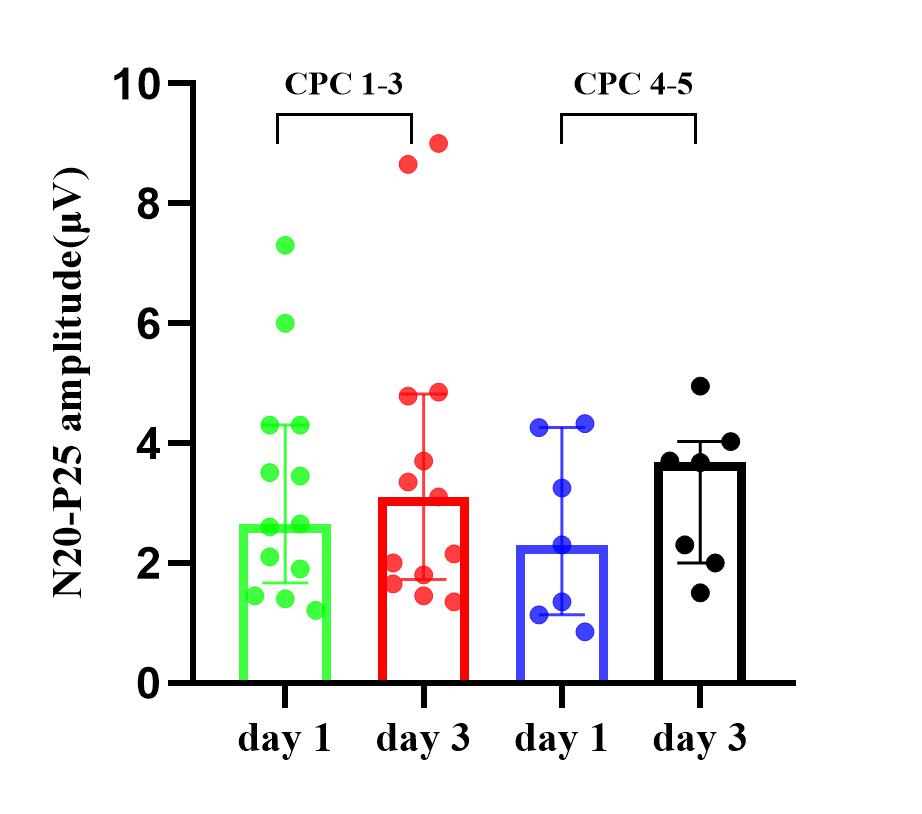


**Fig. S2.** Scatterplots of N20-P25 amplitude and CPC score for patients with two SSEP data. 20 patients had SSEP on the first and third days of ICU admission.13 patients had CPC 1-3 and 7patients had CPC

4-5.


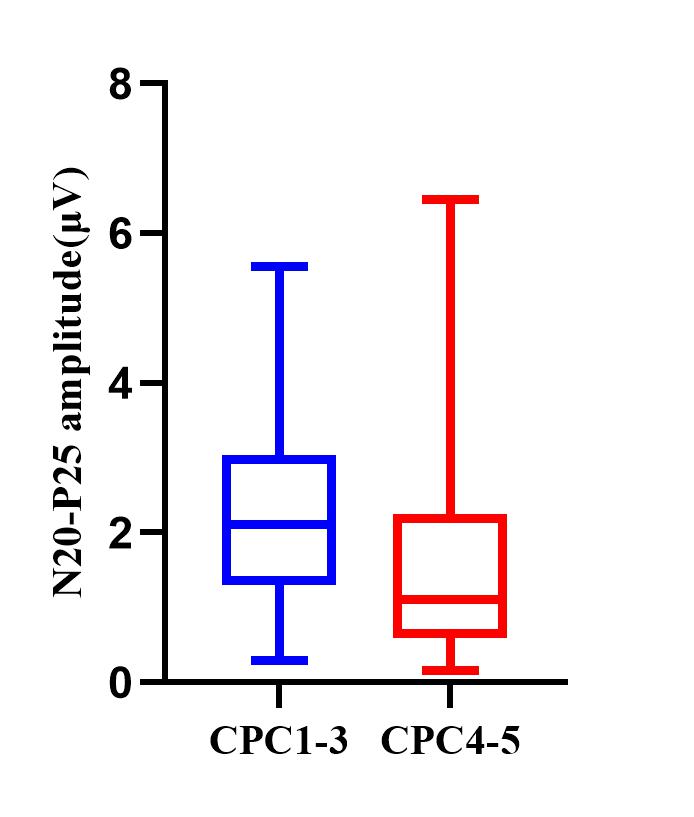


**Fig. S3.** The relationship between the mean value of N20-P25 amplitude and the CPC score for patients with unilateral N20 present(A/P). The median N20-P25 amplitude of CPC 1-3 group is 2.05μV,and the median N20-P25 amplitude of CPC 4-5 group is 0.85μV, there was a statistically significant difference in N20-P25 amplitude between the two groups (*p*<0.001).
