# Supplementary material for: N20-P25 Amplitude can Predict Awakening from Coma
Source: Neurocrit Care. 2025 Aug 11;43(3):902–10. doi: 10.1007/s12028-025-02335-9 (PMC12647323; doi:10.1007/s12028-025-02335-9)
Supplement: Supplementary file 2 — Supplementary file2 (DOCX 20 KB) [file 12028_2025_2335_MOESM2_ESM.docx]

**Table S1** Sensitivity and specificity of N20-P25 amplitude

|  | CPC 1-3 | CPC 4-5 | CPC 4-5 | | CPC 1-3 | |
| --- | --- | --- | --- | --- | --- | --- |
|  |  |  | Sensitivity % (95% CI) | Specificity % (95% CI) | Sensitivity % (95% CI) | Specificity % (95% CI) |
| All(n=420) | (n=190) | (n=230) |  |  |  |  |
| ＜0.88μV, n(%) | 4(2.1) | 42(18.3) | 18.3(13.8-23.8) | 97.8(94.5-99.2) |  |  |
| ＞4.53μV, n(%) | 56(29.5) | 46(20.0) |  |  | 29.5(23.5-36.3) | 80.0(74.4-84.7) |
| TBI(n=62) | (n=24) | (n=38) |  |  |  |  |
| ＜1.2μV, n(%) | 0(0.0) | 13(34.2) | 34.2(21.2-50.1) | 100(86.2-100.0) |  |  |
| ＞4.54μV, n(%) | 11(45.8) | 7(18.4) |  |  | 45.8(27.9-64.9) | 81.6(66.6-90.8) |
| ＞1.2μV, n(%) | 24(100) | 25(65.8) |  |  | 100(86.2-100.0) | 34.2(21.2-50.1) |
| aSAH(n=108) | (n=58) | (n=50) |  |  |  |  |
| ＜0.74μV, n(%) | 0(0.0) | 8(16) | 16(8.3-28.5) | 100(93.8-100.0) |  |  |
| ＞3.64μV, n(%) | 28(48.3) | 10(20.0) |  |  | 48.3(35.9-60.8) | 80(67.0-88.9) |
| ＞0.74μV, n(%) | 58(100) | 42(84) |  |  | 100(93.8-100.0) | 16(8.3-28.5) |
| ICH(n=67) | (n=39) | (n=28) |  |  |  |  |
| ＜0.66μV, n(%) | 0(0.0) | 4(14.3) | 14.3(5.7-31.5) | 100(91.0-100.0) |  |  |
| ＞3.89μV, n(%) | 14(35.9) | 4(14.3) |  |  | 35.9(22.7-51.6) | 85.7(68.5-94.3) |
| ＞0.65μV, n(%) | 39(100) | 24(85.7) |  |  | 100(91.0-100.0) | 14.3(5.7-31.5) |
| CA(n=62) | (n=17) | (n=45) |  |  |  |  |
| ＜1.12μV, n(%) | 0(0.0) | 16(35.6) | 35.6(23.2-50.2) | 100(81.6-100.0) |  |  |
| ＞4.7μV, n(%) | 4(23.5) | 6(13.3) |  |  | 23.5(10.0-47.3) | 86.7(73.8-93.7) |
| ＞1.6μV, n(%) | 17(100) | 24(53.3) |  |  | 100(81.6-100) | 46.7(30.9-60.9) |
